# In Vitro Approbation of Microbial Preparations to Shield Fruit Crops from Fire Blight: Physio-Biochemical Parameters

**DOI:** 10.3390/plants13111431

**Published:** 2024-05-22

**Authors:** Asil A. Nurzhanova, Aigerim Mamirova, Valentina Mursaliyeva, Asiya S. Nurmagambetova, Zhadyra Zhumasheva, Timur Turdiyev, Svetlana Kushnarenko, Elvira Ismailova

**Affiliations:** 1 Institute of Plant Biology and Biotechnology, Timiryazev 45, Almaty 050040, Kazakhstan; gen_asil@mail.ru (A.A.N.); gen_mursal@mail.ru (V.M.); asiyanurm@mail.ru (A.S.N.); zhoomash.zh@mail.ru (Z.Z.); turdievtt@mail.ru (T.T.); svetlana_bio@mail.ru (S.K.); 2 Department of Biotechnology, Faculty of Biology and Biotechnology, Al-Farabi Kazakh National University, Al-Farabi 71, Almaty 050040, Kazakhstan; 3 Scientific Production Centre of Microbiology and Virology, Bogenbai Batyr 105, Almaty 050010, Kazakhstan; elya7506@mail.ru

**Keywords:** biopreparation, fire blight, protein synthesis, antioxidant enzymes, photosynthetic pigments

## Abstract

The need for the increasing geographical spread of fire blight (FB) affecting fruit crops to be addressed led to large-scale chemicalization of the environmental matrices and reduction of plant productivity. The current study aimed to assess the effects of novel biopreparations at different exposure durations on photosynthetic pigment content and antioxidant enzyme activity in leaves of apple and pear varieties with varying levels of resistance to FB. Biopreparations were formulated from a cultural broth containing *Lacticaseibacillus paracasei* M12 or *Bacillus amyloliquefaciens* MB40 isolated from apple trees’ phyllosphere. Aseptic leaves from blight-resistant (endemic *Malus sieversii* cv. KG10), moderately resistant (*Pyrus pyraster* cv. Wild), and susceptible (endangered *Malus domestica* cv. Aport and *Pyrus communis* cv. Shygys) varieties were employed. The impact of biopreparations on fruit crop antioxidant systems and photosynthetic apparatuses was investigated in vitro. Study results indicated that FB-resistant varieties exhibit enhanced adaptability and oxidative stress resistance compared to susceptible ones. Plant response to biopreparations varied based on the plant’s initial FB sensitivity and exposure duration. Indeed, biopreparations improved the adaptive response of the assimilation apparatus, protein synthesis, and catalase and superoxide dismutase activity in susceptible varieties, suggesting that biopreparations have the potential for future commercialization to manage FB in fruit crops.

## 1. Introduction

Fire blight, a severe bacterial disease caused by *Erwinia amylovora (E. amylovora)*—originating from North America—affects over 200 species of both cultivated and wild plants, primarily within the *Rosaceae* family [1]. This disease’s range is expanding annually, with presence reported in at least 46 countries worldwide [2]. In the orchards of southeastern Kazakhstan, bacterial necrosis has been identified since the 1980s, mainly impacting pear and stone fruit trees. The pathogen was not considered a significant threat until 2010, when its spread began posing a considerable risk to wild apple (*Malus sieversii*) forests. By 2013, fire blight was designated as a quarantine disease in the fruit-growing zones for apples (*Malus* spp.) and pears (*Pyrus* spp.) in the south and southeast of Kazakhstan [3,4]. The disease impacts all parts of the tree; infected flowers turn dark brown, and as the bacterial infection advances, the leaves wither, darken, and remain attached to the branches, giving the tree a fire-blighted appearance—hence the name “blight” [5]. Jock et al. [6] highlighted that the primary means of fire blight’s spread to numerous countries is through the international trade of planting materials for fruit and ornamental trees belonging to the *Rosaceae* family. Indeed, from 2008 to 2015, Kazakhstan imported more than 10 million seedlings and rootstocks of apple, pear, and quince [7], indicating a significant pathway for the introduction and dissemination of the disease.

The occurrence of fire blight in plants is influenced by spring weather conditions, species resistance, and age, leading countries to develop tailored control strategies. Traditional methods to manage fire blight include planting resistant varieties, employing agricultural best practices, and chemical treatments with antibiotics and fungicides [8]. However, widespread chemical use in agriculture has led to environmental pollution, reduced plant productivity, and increased resistance of phytopathogens to antibiotics and fungicides [9]. For instance, streptomycin sulfate is effective against fire blight but is banned in many countries [10].

Biological control methods offer a sustainable alternative, utilizing biocontrol agents against *E. amylovora*. Several effective bacterial antagonists of *E. amylovora* have been identified, leading to the development of biopesticides for seed inoculation or plant spraying during the growing season. Notable examples include BlightBan™ A506, based on *Pseudomonas fluorescens* A506 isolated from pear trees’ leaves; BlightBan™ C9-1, based on *Pantoea vagans* C9-1 isolated from apple stems; and Blossom Protect™, formulated based on *Aureobasidium pullulans* isolated from apple leaves [11,12,13,14]. The success in biological control has spurred further research into potential bacterial and fungal biocontrol agents against various crop pests [15,16] and diseases [17]. *Streptomyces* C1-4, for example, have shown promise in reducing fire blight damage; specifically, strain notably decreased symptoms and reduced the incidence of fire blight on leaves by about 70% after two treatments [18]. In Egypt, spraying with non-pathogenic bacteria such as *Bacillus subtilis*, *Pantoea agglomerans*, and Harmel plant extract (*Peganum harmala* L.) was effective in controlling fire blight [19]. Similarly, a talc-based formulation of *Pantoea agglomerans* Eh-24 significantly reduced (by 63–76%) the incidence of diseased flowers in two Turkish pear orchards [20].

Kazakhstan, as well as other countries, is developing programs to protect apple and pear orchards from fire blight, emphasizing an integrated approach that includes sanitation, growth regulators, agricultural techniques, and both biological and chemical measures. These programs follow the main objectives to eliminate infection sources, prevent or reduce pathogen populations, and enhance tree resistance to bacterial infections [21].

Inoculating plants with biopreparations containing live microorganisms is a critical approach to enhancing plant health, making the plant’s response to these products a vital aspect of agricultural research. Plants possess the ability to detect biotic stress and activate regulatory or transcriptional mechanisms to generate appropriate responses. While plant defense mechanisms against pathogens are well-documented, the detailed interaction and impact of various signals in eliciting defense responses to biotic stress are not fully understood [22]. Plants use various defense strategies to cope with these unfavorable factors, and among them, the production of reactive oxygen species (ROS) plays a key role. These mechanisms evolve from constant plant–pathogen interactions [23]. The production of ROS in response to biotic and abiotic stress can damage proteins, nucleic acids, and lipids, leading to oxidative stress. Plants counteract this stress through an innate defense mechanism involving various antioxidant enzymes [24,25].

In vitro tissue culture, believed to be the most suitable model system for testing microbial preparations, provides a controlled environment, allowing for precise manipulation of exogenous factors [26]. Furthermore, probiotic bacterium *Lacticaseibacillus paracasei* exhibits substantial growth-inhibitory effects against various microbes, including *Staphylococcus aureus*, *Escherichia coli*, *Pseudomonas aeruginosa*, *Penicillium chrysogenum*, *Aspergillus niger* [27], and *Propionibacterium acnes* [28]. Additionally, screening for lactic acid bacteria strains’ antagonistic activity against *E. amylovora* identified *Lacticaseibacillus paracasei* M12 as having a significant inhibitory effect on the pathogen’s growth [29].

Therefore, the current research was focused on examining the effects of biopreparations based on *Lacticaseibacillus paracasei* or *Bacillus amyloliquefaciens*, which have demonstrated antagonistic activity against *E. amylovora*, to support and improve the morpho-physiological state of apple and pear trees susceptible or resistant to fire blight, propagated in vitro, evaluating photosynthetic pigment content and antioxidant enzyme activity in leaves. In fact, the study aimed at assessing biopreparations for plant growth-promoting and stress-reducing properties, since microbial strains intended for use as biocontrol agents against phytopathogens should possess multifunctional properties beyond just antagonism. 

## 2. Results

### 2.1. Influence of Biopreparations on the Physiological Parameters of Apple and Pear Micro-Shoots In Vitro

**Figure 1:**
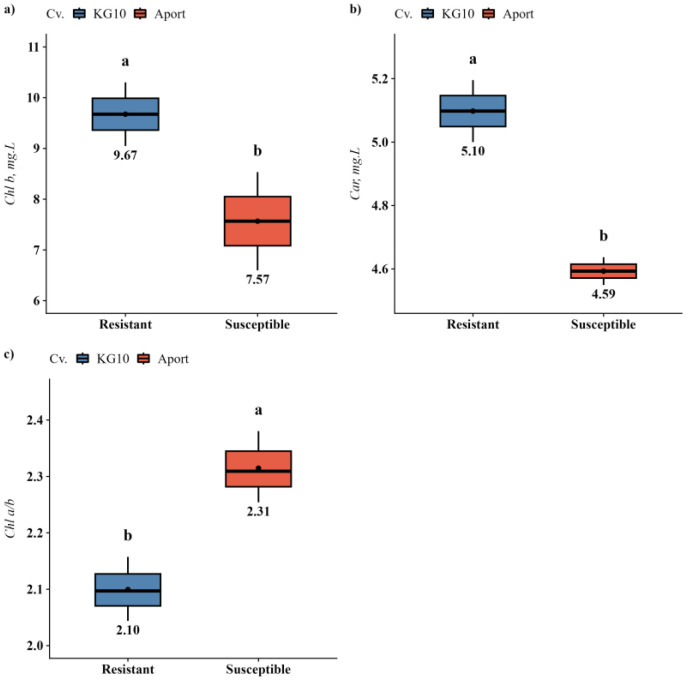
Photosynthetic pigment content in resistant and susceptible apple varieties grown under in vitro conditions: (**a**) *Chl b*; (**b**) *Car*; (**c**) *Chl a/b.* Only parameters with significant differences detected were shown. Different letters within one parameter indicate significant difference.

Investigations into the effects of biopreparations on the photosynthetic apparatus of apple and pear varieties, compared by their susceptibility to fire blight, revealed significant variations only between apple varieties (Table S1). Specifically, the blight-resistant cv. KG10 exhibited a higher concentration of auxiliary pigments, namely chlorophyll *b* (27.8%) and carotenoids (11.0%), in contrast to the susceptible cv. Aport. Thus, this was accompanied by a notable decrease in the chlorophyll *a* to *b* ratio (9.33%) (Figure 1).

Biopreparations significantly influenced the photosynthetic apparatus of apple and pear varieties susceptible to bacterial blight. A notable trend emerged: in blight-susceptible varieties (cv. Aport and cv. Shygys), chlorophyll *b* and consequently the ratio of the sum of chlorophyll *a* and *b* to carotenoids increased, whereas in blight-resistant varieties (cv. KG10 and cv. Wild), only an increase in chlorophyll *a* content was observed (Table 1 and Table 2). Stress bioindicators, such as the increased proportion of auxiliary pigments (chlorophyll *b* and carotenoids), were affected by the exposure time and type of biopreparation. Indeed, no changes in the photosynthetic apparatus were detected under the influence of biopreparations in blight-resistant cv. KG10.

BP1, with prolonged exposure (60 min), increased the content of chlorophyll *a* by 35.7%, chlorophyll *b* by 124%, and the ratio of total chlorophylls to carotenoids by 166% in the blight-susceptible cv. Aport, while reducing the chlorophyll *a* to *b* ratio and carotenoid content by 38.8 and 39.7%, respectively (Table 2). BP2 influenced photosynthetic activity in the leaves of the blight-susceptible cv. Aport regardless of exposure duration (30 or 60 min), showing an increase in chlorophyll *a* (by 22.9–27.0%), chlorophyll *b* (by 84.9–93.1%), and the ratio of total chlorophylls to carotenoids (by 38.8–39.2%) compared to control (water-inoculated micro-shoots). Moreover, a reduction in the chlorophyll *a* to *b* ratio to 31.2–36.3% compared to control was observed (Table 2).

Unlike the blight-resistant apple variety cv. KG10, moderately resistant pear variety cv. Wild exhibited weak resistance to fire blight (Table 1). Under the influence of BP1 with prolonged exposure, the wild pear mirrored the trend observed in the blight-susceptible apple variety cv. Aport, except for a 13.9% increase in the ratio of chlorophyll *a* to *b* relative to the control (Table 1). In contrast to apple varieties, blight-susceptible cv. Shygys treated with BP2 for 30 min showed an increase in chlorophyll *b* content and the ratio of chlorophylls to carotenoids of 57.0 and 35.0%, respectively (Table 2). Conversely, treating blight-susceptible cv. Shygys with BP1 for 60 min reduced chlorophyll *a* and *b* and carotenoid contents by 21.7, 25.2, and 18.6%, respectively (Table 2).

Summarizing the results obtained, it can be concluded that in contrast to BP1, BP2 more effectively enhances the adaptive response of the assimilation apparatus in apple and pear leaves. It was observed that BP1 can influence the photosynthetic apparatus of studied fruit crops only under prolonged exposure (60 min), whereas BP2 begins to affect the above parameter within the 30 min inoculation.

### 2.2. Influence of Biopreparations on the Biochemical Parameters of Apple and Pear Micro-Shoots In Vitro

The study uncovered that the total antioxidant capacity varied not only between resistant and susceptible varieties of pear and apple plants but also among varieties within the same species. Additionally, species-specific differences in the activity of individual enzymes were observed in control samples of the test plants. A notable characteristic of fire blight-resistant apple cv. KG10 was the high activity of superoxide dismutase (SOD), ascorbate peroxidase (APX), and glutathione reductase (GR) (Table 3). Conversely, moderately resistant pear cv. Wild was distinguished by significant catalase (CAT) activity (Table 3).

In control samples, the apple varieties demonstrated notably higher SOD activity in leaves compared to the pear varieties, specifically: blight-resistant cv. KG10 had a SOD activity of 4187 ± 97.7 µmol min^−1^ mg^−1^ protein, being 19 times higher than the enzyme activity in pear varieties; blight-susceptible cv Aport-708 ± 28.2 µmol min^−1^ mg^−1^ protein, being more than 3 times higher (Table 3). CAT activity, prevalent in young viable tissues and organs, was significantly higher in the leaves of pear varieties (cv. Wild—2003 ± 155 µmol min^−1^ mg^−1^ protein, cv. Shygys—1742 ± 88.5 µmol min^−1^ mg^−1^ protein) compared to apple varieties (cv. KG10—6.30 ± 0.77 µmol min^−1^ mg^−1^ protein, cv. Aport—2.60 ± 0.48 µmol min^−1^ mg^−1^ protein), potentially due to the slower growth rate of pear varieties under in vitro conditions.

APX activity in the blight-resistant cv. KG10 (51.9 ± 4.66 µmol min^−1^ mg^−1^ protein) was higher than in the leaves of blight-susceptible cv. Aport (9.50 ± 1.20 µmol min^−1^ mg^−1^ protein) by 81.7% (Table 4). APX activity in the leaves of moderately resistant cv. Wild was twice lower than in the leaves of blight-resistant cv. KG10, whereas the blight-susceptible cv. Shygys exhibited 2.3 times higher enzyme activity than the blight-susceptible cv. Aport. APX activity did not essentially (by 13.0%) differ between the pear varieties (cv. Wild—26.2 ± 1.54 µmol min^−1^ mg^−1^ protein, cv. Shygys—21.9 ± 1.01 µmol min^−1^ mg^−1^ protein), indicating structural and functional peculiarities of the plant and a potential increase in H_2_O_2_ concentration, leading to enzyme activation [30].

Under control conditions, GR activity in the leaves of blight-resistant apple and pear varieties was higher by 61.6 and 45.4% compared to the blight-susceptible varieties, respectively (Table 3). The results suggest that the blight-resistant apple variety cv. KG10 shows significantly increased antioxidant enzymes’ activity—up to 5.91 times—compared to the blight-susceptible apple variety cv. Aport. In contrast, the moderately resistant pear variety cv. Wild exhibited enhanced CAT activity. Furthermore, blight-resistant varieties displayed reduced protein synthesis compared to blight-susceptible varieties. Therefore, it can be concluded that blight-susceptible varieties can be characterized by increased protein synthesis (by 318 and 35% in relation to resistant ones), indicating a potential physiological response to stress conditions (Table 3).

The response of SOD activity to treatment with BP1 varied based on the plant resistance level and the exposure duration (Table 4 and Table 5). A 30 min and 60 min inoculation increased SOD activity by 24.8 and 70.0%, respectively, in the leaves of the blight-susceptible apple variety cv. Aport (Table 5). Conversely, in the blight-resistant apple variety cv. KG10, SOD activity decreased by 13.6 and 46.6% after 30 and 60 min exposure, respectively, compared to the control (Table 4). For pear varieties, the response of SOD activity to treatment also depended on their susceptibility. In the leaves of blight-susceptible cv. Shygys, a significant decrease of 75.3 and 90.6% within 30 and 60 min exposure was observed (Table 5). In contrast, the blight-resistant cv. Wild showed an increase in SOD activity by 549% after 30 min exposure but a decrease of 91.6% after 60 min exposure compared to the control (Table 4).

Inoculation of studied plants with BP2 for 30 and 60 min resulted in a decrease in SOD activity by 84.2–87.5% in the blight-resistant cv. KG10, whereas no effect was observed in the case of blight-susceptible cv. Aport (Table 4 and Table 5).

As for the pear varieties, BP2 increased SOD activity in the leaves of moderately resistant cv. Wild and blight-susceptible cv. Shygys by 233–267 and 12.2% (60 min exposure), respectively (Table 4 and Table 5). Thus, the above changes in SOD activity suggest an association with the plants’ response to increased ROS levels in the cells induced by the biotic stressor, potentially enhancing their stress resistance.

CAT activity in the leaves of the blight-resistant cv. KG10 increased significantly upon treatment with BP1, showing increases of 105 and 92.6% for 30 and 60 min exposure, respectively (Table 4). In contrast, the blight-susceptible cv. Aport exhibited an increase in CAT activity proportional to exposure time, with increases of 98.9 and 189%, respectively, relative to the control (Table 5). BP2 induced a remarkable stimulation in CAT activity in both resistant and susceptible apple varieties (up to 713 times).

In the case of pear varieties treated with BP1, the CAT activity in cv. Wild increased by 108% after a 30 min exposure but decreased by 59.3% after a 60 min exposure (Table 4). For the blight-susceptible cv. Shygys, CAT activity remained at the control level under 30 min exposure and increased by 209% when exposed to BP1 for 60 min (Table 5). Similarly, inoculation of pear varieties with BP2 for 30 min increased CAT activity by up to 67.6%, whereas inoculation for 60 min decreased enzyme activity by up to 48.2% (Table 4 and Table 5).

Regarding the APX activity, treatment with BP1 for 30 min resulted in a 39.6% increase in the leaves of cv. Wild; however, with prolonged exposure of 60 min, enzyme activity decreased by 34.4% relative to the control (Table 4). There was no observed effect of BP1 on APX activity in the leaves of the blight-resistant apple variety cv. KG10 and blight-susceptible pear variety cv. Shygys. Indeed, an increase in APX activity was detected in the leaves of the blight-susceptible apple variety cv. Aport, when exposed to BP1 for 60 min, by 114% relative to the control (Table 5).

Treatment with BP1 led to an increase in GR activity across studied varieties. Specifically, in the fire blight-resistant apple variety cv. KG10, GR activity increased by 62.8–107% regardless of the exposure duration (Table 4). The blight-susceptible pear variety cv. Shygys showed increases of 69.9 and 135% relative to the control for different exposure duration of BP1 (Table 5). However, the moderately resistant pear variety cv. Wild experienced an increase in GR activity of 88.2% relative to the control only after a 30 min inoculation with BP1. Conversely, treatment with BP2 resulted in a decrease in GR activity in the leaves of all studied varieties up to 93.2% relative to the control, except for the cv. Shygys, which exhibited a 94.9% increase at 60 min exposure relative to the control (Table 4 and Table 5).

Thus, the above results can be summarized as follows: (1) tested biopreparations increased the CAT activity in the leaves of blight-resistant varieties depending on exposure duration; (2) inoculation with BP1 increased the activity of protein synthesis and antioxidant enzymes in the leaves of moderately resistant pear variety cv. Wild at a 30 min exposure but led to a decrease at 60 min exposure relative to the control; (3) inoculation with BP2 increased the protein synthesis and reduced GR activity in the leaves of blight-resistant and susceptible apple varieties; (4) treatment with BP2 regardless of exposure duration reduced the GR and APX activity and increased SOD activity in the leaves of moderately resistant pear variety cv. Wild, moreover, BP2 maintained the same pattern for APX and SOD activity in the leaves of blight-susceptible pear variety cv. Shygys; however, for the latter enzyme–only at 60 min exposure.

## 3. Discussion

Study findings indicated that inoculation of aseptic cultures with cultural broths based on *Lacticaseibacillus paracasei* (BP1) or *Bacillus amyloliquefaciens* (BP2) with the antagonistic activity against the pathogen *E. amylovora* did not significantly influence the micro-shoots morphological parameters in vitro. Nevertheless, minor variations were observed in the elongation of new shoots during development, as indicated by changes in the reproduction coefficient. Notably, the duration of exposure to BP2, unlike BP1, impacted the formation of new shoots during morphogenesis. A 30 min exposure increased the reproduction rate by up to 70% in apple and pear varieties resistant to the fire blight. Conversely, in the susceptible apple variety, the reproduction rate decreased by 36% compared to the control group, regardless of the exposure duration.

The innate defense mechanisms of plants are largely predicated on the synthesis of both enzymatic and non-enzymatic antioxidants [31]. The current study focused on evaluating the impact of newly developed biopreparations, formulated as cultural broths and based on live microorganisms *L. paracasei* M12 or *B. amyloliquefaciens* MB40 isolated from the apple phyllosphere, on photosynthetic pigment content and antioxidant enzyme activity in the leaves of fruit crops that are either resistant or susceptible to fire blight under in vitro conditions. It was determined that photosynthetic pigment content undergoes significant and complex changes under the influence of tested biopreparations and varies depending on the plants’ resistance or susceptibility to fire blight. *L. paracasei* M12 (BP1) is a novel species used against fire blight in orchard trees with no evidence published except for our recent study [29]. On the other hand, *B. amyloliquefaciens* strains were widely tested on the ability to mitigate the *E. amylovora*-induced disease in fruit crops [32,33,34,35,36,37,38,39,40,41,42]. Genome analysis of *B. amyloliquefaciens* FZB42 mutant strains blocked in production of difficidin (CH8 Δ*dfn*), polyketides (CH3 Δ*sfp*), and polyketide and bacilysin (RS06 Δ*sfp* Δ*bac*) reported that inhibitory effect of *B. amyloliquefaciens* strains in relation to growth of *E. amylovora* can be explained by the bacilysin synthesis [32]. In addition, a secondary metabolite oxydifficidin, produced by *B. amyloliquefaciens* ssp. plantarum FL50S, was found to be most efficient against *E. amylovora* S435 [39].

Furthermore, a study on the antagonistic activity of *B. amyloliquefaciens* MB40 (BP2) against *E. amylovora* 1E IMIV in the bioassay of immature pear plants under in vitro conditions revealed that strain efficiency reached up to 90.6% [33]. A two-year field trial consisted of spray treatments with different bacterial antagonists on susceptible apple cultivars ‘Gala’, ‘Golden Parsi’, and ‘Golden Smoothee’ identified *B. amyloliquefaciens* LMR2 (isolated from apple blossom) as a perspective biocontrol agent for ‘Golden Parsi’ cultivar with disease control efficacy reaching 100%, while B. amyloliquefaciens SP18 (isolated from soil) for all three cultivars, with disease control efficacy ranging from 59.6 to 88.9% [34].

Investigating the impact of biopreparations on different apple and pear varieties, distinct responses were noted. For a resistant apple variety cv. KG10, exposure to biopreparations resulted in a reduction in carotenoid levels without affecting chlorophyll *a* and *b* contents (Table 2). Conversely, in the susceptible apple variety cv. Aport, an increase in chlorophyll *a* and *b* content was observed alongside a decrease in carotenoid levels. The response of a moderately resistant pear variety cv. Wild to biopreparations was as follows: an increase in chlorophyll *a* and *b* content was observed across all treatments and a decrease in carotenoids when exposed to BP1. In the susceptible pear variety cv. Shygys, both chlorophyll and carotenoid content decreased, regardless of the biopreparations. Iwaniuk and Lozowicka [43] reported that biotic stress may reduce chlorophyll content by inhibiting photosynthetic enzymes or disrupting both photosystems I and II. BP2 was found to be more effective in enhancing the adaptive response of the assimilation apparatus in apple and pear leaves compared to BP1 by increasing chlorophyll *b* content and, consequently, the ratio of total chlorophyll to carotenoids. These findings align with existing literature that underscores the importance of chlorophyll *b* and carotenoids (as auxiliary photosynthetic pigments) in mitigating biotic and abiotic stresses [44,45]. The observed increase in auxiliary pigments is indicative of the plants’ adaptive response of the assimilation apparatus to exogenous factors. Furthermore, BP2, as a representative of *Bacillus* sp., was proved to be a promising biocontrol agent due to the capability of enhancing plant defense through induced systemic resistance, improving nutrient availability, altering plant growth hormones homeostasis, and reducing abiotic stress apart from antagonistic mechanisms against phytopathogens [46].

Biotic and abiotic stresses induce the formation of ROS in plants, which can cause destructive effects on plant tissues and cells. In response to the accumulation of ROS, plants activate antioxidant defense systems [24,47], deploying system-protective strategies to eliminate excess ROS and maintain cellular redox homeostasis during oxidative stress [31]. Research findings particularly highlight the effectiveness of the BP2 based on *B. amyloliquefaciens* MB40 in enhancing CAT activity in apple trees at 30 min exposure compared to BP1 based on *L. paracasei* M12, being in alignment with literature data on the active role of the enzyme in plant growth and development [48].

Plant inoculation with biopreparations based on *L. paracasei* M12 (BP1) or *B. amyloliquefaciens* MB40 (BP2) led to several notable outcomes. In the leaves of blight-resistant apple variety cv. KG10, CAT activity and protein synthesis increased with longer exposure time, while SOD activity decreased. BP2, regardless of exposure duration, reduced APX activity but increased protein synthesis, as well as GR and SOD activities in the blight-susceptible pear variety cv. Shygys. In the leaves of blight-susceptible apple variety cv. Aport, BP2 increased protein synthesis and CAT activity and reduced GR activity. An increase in GR activity signals the activation of glutathione reduction reactions, crucial for neutralizing ROS [49]. Indeed, biotic and abiotic stress factors induce GR activity in plants [50].

BP1 enhanced the activity of SOD, APX, and CAT in the blight-susceptible cv. Aport, positively correlating with exposure duration while reducing GR activity. A 30 min exposure emerged as the optimal duration for BP1, with peak activities of SOD and CAT relative to control. Indeed, this peculiarity suggests a functional interplay between these antioxidant enzymes, where SOD catalyzes the neutralization of the superoxide anion radical into H_2_O_2_, subsequently decomposed by CAT in apple trees and by a CAT and APX complex in pears with minimal BP1 exposure. The increase in SOD activity, a primary antioxidant enzyme, is presumed to be related to the high ROS levels in plant cells in response to biotic stress. Numerous studies have reported a positive correlation between SOD activity under abiotic and biotic stress conditions and plant resistance [31,51,52,53]. Under prolonged exposure, GR activity decreased in apple leaves but increased in the pear variety cv. Shygys, indicating the potential of tested biopreparations to enhance the activity of key enzymes in the plant defence system respective of exposure duration, thereby bolstering plant resistance to oxidative stress. These results corroborate literature data on the activation of antioxidant defense mechanisms in response to abiotic and biotic stress [54].

## 4. Materials and Methods

### 4.1. Research Materials

Biopreparation No. 1 (BP1) in the form of a cultural broth of *Lacticaseibacillus paracasei* M12, isolated in 2022 from the apple phyllosphere [29]. The strain uses arabinose, ribose, cellobiose, galactose, glucose, gluconate, raffinose, maltose, melibiose, sucrose, lactose, sorbitol, dulcitol, mannitol, and mannose as a carbon source; produces lactic and acetic acids. *Lacticaseibacillus paracasei* M12 is a Gram-positive, facultative anaerobe deposited in the REM on the RSE “Republican Collection of Microorganisms” of the Committee of Science of the Ministry of Science and Higher Education of the Republic of Kazakhstan under number B-R-KM 1082.

Biopreparation No. 2 (BP2) in the form of a cultural broth of *Bacillus amyloliquefaciens* MB40, isolated in 2017 from the ‘Zarya Alatau’ apple tree phyllosphere [33]. The strain uses glucose, mannose, fructose, ribose, arabinose, cellobiose, and maltose as a carbon source; produces acetoin; hydrolyses starch, urea, and esculin; has catalase activity. *Bacillus amyloliquefaciens* MB40 is a Gram-positive anaerobe deposited in the REM on the RSE “Republican Collection of Microorganisms” of the Committee of Science of the Ministry of Science and Higher Education of the Republic of Kazakhstan under the number B-R-KM 0846.

**Figure 2:**
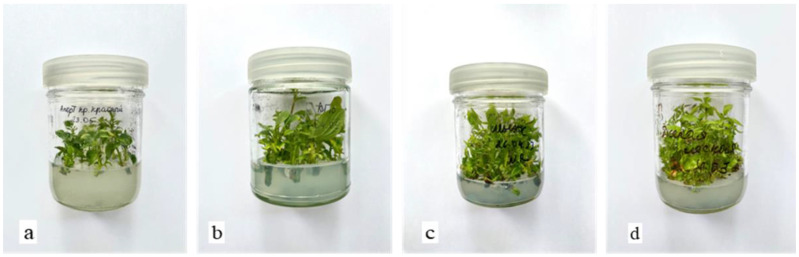
Aseptic plants of apple and pear varieties. (**a**) *M. domestica* cv. Aport; (**b**) *M. sieversii* cv. KG10; (**c**) *P. communis* cv. Shygys; (**d**) *P. pyraster* cv. Wild.

In vitro cultures of aseptic shoots from both apple and pear varieties, exhibiting varying levels of resistance to fire blight (Figure 2): *Malus sieversii* (Ledeb.) M. Roem cv. KG10—resistant; *Malus domestica* Borkh. cv. Aport Blood-red–susceptible; *Pyrus pyraster* L.–moderately resistant; *Pyrus communis* L. cv. Shygys—susceptible. *M. sieversii* cv. KG10 is endangered and endemic in Kazakhstan. Each treatment was performed in 5 replications.

### 4.2. Obtaining In Vitro Cultures of Aseptic Shoots

Apple shoots were cultured on Murashige and Skoog (MS) medium supplemented with 0.5 mg L^−1^ 6-benzylaminopurine (BAP), 0.01 mg L^−1^ indole-3-butyric acid (IBA), and 30 g L^−1^ sucrose at a pH of 5.7. Pear shoots were cultured on MS medium with 0.6 mg L^−1^ BAP, 0.1 mg L^−1^ IBA, 0.2 mg L^−1^ humic acid (HA), and 30 g L^−1^ sucrose at a pH of 5.7. The plants were grown in a controlled environment room under the following conditions: a temperature of 24 °C, a light intensity of 25 µmol m^−2^ s^−1^, and a 16 h photoperiod. In vitro subculturing to fresh medium occurred every 4 weeks, during which the health and proliferation of shoots were monitored. The reproduction coefficient for each subculture cycle was calculated using the following equation:

(1) $$R_{c}=\frac{a}{b}\times c$$

where *a*—the number of shoots formed; *b*—the number of shoots planted (for propagation); *c*—the number of subculturing (passages).

A preliminary investigation on biopreparations’ phytotoxicity determined that the optimal concentration range for treating fruit crops with BP1 and BP2 to address bacterial blight is between 0.5 and 5.0% [own data]. Micro-shoots of apple and pear varieties were inoculated with these biopreparations at a concentration of 1% for 30 and 60 min under in vitro conditions. Water-inoculated micro-shoots of apple or pear varieties served as control. Physiological and biochemical analyses were conducted 1 month following the inoculation; each analysis was performed in 3 biological and 3 chemical repetitions.

### 4.3. Determination of Chlorophyll Pigments’ Content

The concentration of chlorophyll (*Chl a* and *Chl b*) and carotenoids (*Car*) were determined in leaves of aseptic cultures of apple and pear varieties, according to Gavrilenko et al. [55]. Then, 30 mg of aseptic culture leaves was sampled and crushed in 2 mL of chilled 96% ethanol; the resulting homogenate was centrifuged for 10 min at 7000 rpm. The supernatant was poured into a test tube, and the absorbance of photosynthetic pigments was determined at 440.5, 649, and 665 nm, consequently, using spectrophotometer Evolution 60 (Thermo Scientific, Waltham, MA, USA). Equations used for calculating *Chl a*, *Chl b*, and *Car* concentrations were shown in detail [55,56].

### 4.4. Determination of Antioxidant Enzyme Activity

Aseptic culture leaves (0.2 g FW) were ground with quartz sand in a mortar before being re-suspended in 1.2 mL of 0.2 M Na-K phosphate buffer solution (pH = 6.0). The homogenate was centrifuged for 15 min at 4 °C at 9000 rpm. A clean tube was used to collect the supernatant. After centrifugation, the precipitate was dissolved in 0.8 mL of 0.2 M Na-K phosphate buffer solution (pH 6.0). The first and second portions of supernatant were mixed and used as an enzyme extract for catalase (CAT; EC 1.11.1.6), ascorbate peroxidase (APX; EC 1.11.1.11), glutathione reductase (GR; EC 1.6.4.2), and superoxide dismutase (SOD; EC 1.15.1.1). The enzyme activity and protein content were determined using an SF-2000 spectrophotometer (OKB Spectr, St. Petersburg, Russia).

The protein concentration of enzyme extracted from leaves was determined at λ = 595 nm according to Bradford [57]. A calibration curve was constructed to obtain a linear trendline equation (y = 0.0467*x* − 0.0037; R^2^ = 0.9926) for calculating protein concentration [57]. The activity of CAT, APX, and GR was calculated based on Beer’s–Lambert law [58], while the activity of SOD was determined according to Fazlieva et al. [59]. A detailed description of the protocols and formulas used to determine the protein synthesis and antioxidant enzyme activity was shown in Nurzhanova et al. [56].

### 4.5. Statistical Data Processing

The data analysis was conducted using RStudio software (version 2023.06.0 Build 421, RStudio PBC, 2023). Tukey HSD tests were performed for the pairwise comparisons of the means, while ANOVA was used to confirm statistical significance. In the case where Shapiro–Wilk test failed, non-parametric Kruskal–Wallis’s test was applied to determine the significant difference, followed by pairwise comparison with ‘Bonferroni’ adjustment. Subsequently, the treatments were categorized by letter in descending order, and graphs were generated. Significance was declared at *p* < 0.05.

## 5. Conclusions

Fire blight-resistant wild apple and pear varieties exhibited higher activities of antioxidant enzymes and increased content of photosynthetic pigments compared to susceptible ones, demonstrating their superior adaptability and resistance to oxidative stress. Notably, the fire blight-resistant apple variety cv. KG10 displayed elevated activities of SOD, APX, and GR (4187 ± 97.7, 51.9 ± 4.66, and 2.70 ± 0.45 µmol min^−1^ mg^−1^ protein, respectively), alongside an increased content of chlorophyll *a* (20.3 ± 0.77 mg mL^−1^). In contrast, the moderately resistant pear variety cv. Wild showed enhanced activities of CAT (2003 ± 155 µmol min^−1^ mg^−1^ protein).

The study revealed that plant responses to biopreparations were influenced by the specific type of product tested, the plants’ inherent susceptibility to fire blight, and the exposure duration. BP2, unlike BP1, was found to increase protein synthesis in the leaves of both resistant and susceptible varieties of apple and pear up to 541%. The activity of antioxidant enzymes SOD and CAT in response to oxidative stress was observed to increase with the exposure duration, underscoring a positive correlation between the activities of SOD, GR, and CAT under biotic stress conditions and plant resistance.

Furthermore, it was determined that a 30 min exposure to BP2 significantly boosts the adaptive response of the assimilation apparatus in susceptible apple and pear varieties. Indeed, 30 min inoculation with BP2 enhances protein synthesis (up to 44%) and the activities of CAT (up to 355 times) more effectively compared to BP1. A 60 min exposure was identified as the optimal treatment time for BP1, leading to increased activities of APX (up to 114%), CAT (up to 189%), and SOD (up to 70%), which signifies an improved plant adaptation to biotic stress. Further research should focus on direct inoculation of studied fruit trees with *E. amylovora* to validate the efficacy of these biopreparations against fire blight.

## Figures and Tables

**Table 1:** Chlorophyll pigments and carotenoid content in blight-resistant apple and pear varieties (mg mL^−1^). Different letters (a, b, c) within one parameter of specific cultivar show the significant difference.

Parameter	Cv.	BP	Ctrl		Time of Seed Inoculation, min				*p*-Value (n = 9)
					30	% to Ctrl	60	% to Ctrl	
*Chl a*	*M. sieversii* cv. KG10	No. 1	20.3 ± 0.77	−	19.2 ± 0.73	−	19.1 ± 0.75	−	0.178
		No. 2		−	19.6 ± 0.96	−	20.3 ± 0.68	−	0.563
	*P. pyraster* cv. Wild	No. 1	15.1 ± 2.11	b	12.3 ± 0.99 b	81.6	20.1 ± 0.97 a	133	<0.01
		No. 2		b	16.4 ± 0.07 ab	108	18.6 ± 0.13 a	123	<0.05
*Chl b*	*M. sieversii* cv. KG10	No. 1	9.67 ± 0.63	−	8.89 ± 0.68	−	8.92 ± 0.37	−	0.245
		No. 2		−	10.2 ± 0.58	−	10.4 ± 0.31	−	0.266
	*P. pyraster* cv. Wild	No. 1	6.34 ± 0.76	b	4.64 ± 0.32 b	73.2	10.2 ± 0.90 a	160	<0.001
		No. 2		c	7.59 ± 0.05 b	120	8.81 ± 0.06 a	139	<0.01
*Car*	*M. sieversii* cv. KG10	No. 1	5.10 ± 0.10	ab	5.12 ± 0.04 a	100	4.92 ± 0.08 b	96.4	<0.05
		No. 2		a	4.86 ± 0.21 a	95.3	4.41 ± 0.05 b	86.5	<0.01
	*P. pyraster* cv. Wild	No. 1	5.31 ± 0.08	a	3.86 ± 0.38 c	72.7	5.06 ± 0.10 b	95.3	<0.001
		No. 2		−	5.09 ± 0.11	−	5.38 ± 0.50	−	0.351
*Chl a/b*	*M. sieversii* cv. KG10	No. 1	2.10 ± 0.06	−	2.16 ± 0.08	−	2.14 ± 0.01	−	0.441
		No. 2		−	5.09 ± 0.11	−	5.38 ± 0.50	−	0.185
	*P. pyraster* cv. Wild	No. 1	2.38 ± 0.05	b	2.71 ± 0.05 a	114	2.08 ± 0.20 b	87.5	<0.01
		No. 2		a	2.16 ± 0.02 ab	90.7	2.12 ± 0.02 b	88.9	<0.05
*Chl (a+b)/Car*	*M. sieversii* cv. KG10	No. 1	5.88 ± 0.39	−	5.49 ± 0.33	−	5.70 ± 0.33	−	0.441
		No. 2		b	6.16 ± 0.54 ab	105	6.97 ± 0.30 a	119	<0.05
	*P. pyraster* cv. Wild	No. 1	4.03 ± 0.47	b	4.27 ± 0.03 b	106	5.90 ± 0.59 a	146	<0.01
		No. 2		b	4.71 ± 0.11 ab	117	5.14 ± 0.44 a	127	<0.05

Notes: Cv.—cultivar; BP—biopreparation; Ctrl—control (water-inoculated micro-shoots); *Chl a*—chlorophyll *a*; *Chl b*—chlorophyll *b*; *Car*—carotenoids.

**Table 2:** Chlorophyll pigments and carotenoid content in blight-susceptible apple and pear varieties (mg mL^−1^). Different letters (a, b) within one parameter of specific cultivar show the significant difference.

Parameter	Cv.	BP	Ctrl		Time of Seed Inoculation, min				*p*-Value (n = 9)
					30	% to Ctrl	60	% to Ctrl	
*Chl a*	*M. domestica* cv. Aport	No. 1	17.5 ± 1.76	b	18.9 ± 1.85 b	108	23.7 ± 0.09 a	136	<0.01
		No. 2		b	22.2 ± 0.31 a	127	21.5 ± 1.29 a	123	<0.01
	*P. communis* cv. Shygys	No. 1	16.4 ± 1.40	a	14.8 ± 0.56 ab	89.9	12.9 ± 1.54 b	78.3	<0.05
		No. 2		ab	20.3 ± 2.20 a	124	12.8 ± 1.33 b	78.2	<0.01
*Chl b*	*M. domestica* cv. Aport	No. 1	7.57 ± 0.97	b	8.19 ± 0.63 b	108	17.0 ± 0.25 a	224	<0.05
		No. 2		b	14.0 ± 1.22 a	s185	14.6 ± 1.23 a	193	<0.001
	*P. communis* cv. Shygys	No. 1	6.71 ± 0.86	a	5.77 ± 0.19 ab	85.9	5.02 ± 0.55 b	74.8	<0.05
		No. 2		b	10.5 ± 1.15 a	157	4.53 ± 0.48 b	67.4	<0.001
*Car*	*M. domestica* cv. Aport	No. 1	4.59 ± 0.04	a	4.53 ± 0.06 a	98.5	2.81 ± 0.05 b	61.2	<0.05
		No. 2		b	4.78 ± 0.07 a	104	4.87 ± 0.03 a	106	<0.01
	*P. communis* cv. Shygys	No. 1	4.99 ± 0.34	a	4.29 ± 0.13 b	86.0	4.06 ± 0.29 b	81.4	<0.05
		No. 2		ab	5.21 ± 0.18 a	104	4.27 ± 0.34 b	85.5	<0.05
*Chl a/b*	*M. domestica* cv. Aport	No. 1	2.32 ± 0.06	a	2.15 ± 0.10 a	93.0	1.40 ± 0.02 b	60.3	<0.05
		No. 2		a	1.59 ± 0.12 b	68.8	1.48 ± 0.13 b	63.7	<0.05
	*P. communis* cv. Shygys	No. 1	2.46 ± 0.11	−	2.56 ± 0.02	−	2.56 ± 0.03	−	0.411
		No. 2		ab	2.23 ± 0.30 b	90.6	2.81 ± 0.02 a	114	<0.05
*Chl (a+b)/Car*	*M. domestica* cv. Aport	No. 1	5.44 ± 0.54	b	6.13 ± 0.61 b	113	14.5 ± 0.36 a	266	<0.05
		No. 2		b	7.57 ± 0.38 a	139	7.55 ± 0.37 a	139	<0.01
	*P. communis* cv. Shygys	No. 1	4.64 ± 0.18	−	4.78 ± 0.04	−	4.41 ± 0.21	−	0.077
		No. 2		b	5.95 ± 0.85 a	135	4.06 ± 0.11 b	68.2	<0.01

Notes: Cv.—cultivar; BP—biopreparation; Ctrl—control; *Chl a*—chlorophyll *a*; *Chl b*—chlorophyll *b*; *Car*—carotenoids.

**Table 3:** Antioxidant enzymes’ activity in untreated plants (µmol min^−1^ mg^−1^ protein). Different letters (a, b) within one parameter show the significant difference.

Parameter	Cv. KG10	Cv. Aport	% to Resistant	*p*-Value (n = 9)	Cv. Wild	Cv. Shygys	% to Resistant	*p*-Value (n = 9)
	Resistant	Susceptible			Resistant ^a^	Susceptible		
Protein	0.20 ± 0.03 b	0.84 ± 0.02 a	418	<0.05 *	0.46 ± 0.06 b	0.62 ± 0.05 a	135	<0.05
APX	51.9 ± 4.66 a	9.50 ± 1.20 b	18.3	<0.05 *	26.2 ± 1.54 a	21.9 ± 1.01 b	83.8	<0.05
CAT	6.30 ± 0.77 a	2.60 ± 0.48 b	41.2	<0.01	2 003 ± 155	1 742 ± 88.5	−	0.064
GR	2.70 ± 0.45 a	1.00 ± 0.05 b	38.4	<0.01	1.20 ± 0.02 a	0.60 ± 0.04 b	54.6	<0.05 *
SOD	4187 ± 97.7 a	708 ± 28.2 b	16.9	<0.05 *	218 ± 3.86 b	237 ± 7.67 a	109	<0.05

Notes: Cv.—cultivar; APX—ascorbate peroxidase; CAT—catalase; GR—glutathione reductase; SOD—superoxide dismutase; ^a^—moderately resistant; *—Kruskal–Wallis test.

**Table 4:** Antioxidant enzymes’ activity in blight-resistant apple and pear varieties (µmol min^−1^ mg^−1^ protein). Different letters (a, b, c) within one parameter of specific cultivar show the significant difference.

Parameter	Cv.	BP	Ctrl		Time of Micro-Shoots’ Inoculation, min				*p*-Value (n = 9)
					30	% to Ctrl	60	% to Ctrl	
Protein	*M. sieversii* cv. KG10	No. 1	0.20 ± 0.03	−	0.19 ± 0.01	96.5	0.19 ± 0.01	94.0	0.737
		No. 2		c	0.79 ± 0.09 b	397	1.08 ± 0.11 a	541	<0.001
	*P. pyraster* cv. Wild	No. 1	0.46 ± 0.06	b	0.57 ± 0.05 a	124	0.20 ± 0.01 c	44.0	<0.001
		No. 2		b	0.48 ± 0.12 b	105	1.06 ± 0.13 a	232	<0.001
APX	*M. sieversii* cv. KG10	No. 1	51.9 ± 4.66	−	48.2 ± 3.44	92.9	49.9 ± 4.58	96.1	0.595
		No. 2		a	14.1 ± 1.29 b	27.1	7.45 ± 0.91 b	14.4	<0.001
	*P. pyraster* cv. Wild	No. 1	26.2 ± 1.54	b	36.6 ± 1.79 a	140	17.2 ± 1.90 c	65.6	<0.001
		No. 2		a	5.98 ± 1.11 b	22.8	5.17 ± 0.40 b	19.7	<0.05
CAT	*M. sieversii* cv. KG10	No. 1	6.29 ± 0.77	b	12.9 ± 1.66 a	205	12.1 ± 0.52 a	193	<0.001
		No. 2		c	1 665 ± 40.1 b	265×	2 320 ± 163 a	369×	<0.001
	*P. pyraster* cv. Wild	No. 1	2 003 ± 155	b	4 169 ± 181 a	208	816 ± 62.7 **c**	40.7	<0.001
		No. 2		b	2 968 ± 150 a	148	1 186 ± 165 **c**	59.2	<0.001
GR	*M. sieversii* cv. KG10	No. 1	2.69 ± 0.45	b	4.38 ± 0.97 ab	163	5.55 ± 1.08 a	207	<0.05
		No. 2		a	0.29 ± 0.05 b	10.9	0.18 ± 0.03 b	6.85	<0.01
	*P. pyraster* cv. Wild	No. 1	1.18 ± 0.02	b	2.23 ± 0.23 a	188	0.80 ± 0.10 c	67.5	<0.001
		No. 2		a	0.16 ± 0.02 b	13.8	0.12 ± 0.01 b	9.80	<0.01
SOD	*M. sieversii* cv. KG10	No. 1	4 187 ± 97.7	a	3 616 ± 119 b	86.4	2 236 ± 163 c	53.4	<0.001
		No. 2		a	663 ± 60.4 b	15.8	524 ± 32.3 b	12.5	<0.01
	*P. pyraster* cv. Wild	No. 1	218 ± 3.86	b	1 412 ± 160 a	649	18.2 ± 1.07 c	8.38	<0.01
		No. 2		b	799 ± 164 a	367	724 ± 72.8 a	333	<0.001

Notes: Cv.—cultivar; BP—biopreparation; Ctrl—control; APX—ascorbate peroxidase; CAT—catalase; GR—glutathione reductase; SOD—superoxide dismutase.

**Table 5:** Antioxidant enzyme activity in blight-susceptible apple and pear varieties (µmol min^−1^ mg^−1^ protein). Different letters (a, b, c) within one parameter of specific cultivar show the significant difference.

Parameter	Cv.	BP	Ctrl		Time of Micro-Shoots’ Inoculation, min				*p*-Value (n = 9)
					30	% to Ctrl	60	% to Ctrl	
Protein	*M. domestica* cv. Aport	No. 1	0.84 ± 0.02	a	0.71 ± 0.05 a	84.8	0.46 ± 0.09 b	54.8	<0.001
		No. 2		b	1.20 ± 0.06 a	144	1.19 ± 0.04 a	142	<0.05
	*P. communis* cv. Shygys	No. 1	0.62 ± 0.05	a	0.75 ± 0.07 a	122	0.47 ± 0.04 b	76.0	<0.01
		No. 2		−	0.73 ± 0.06	119	0.74 ± 0.09	119	0.118
APX	*M. domestica* cv. Aport	No. 1	9.50 ± 1.20	b	9.05 ± 1.02 b	95.3	20.4 ± 3.35 a	214	<0.05
		No. 2		−	8.91 ± 1.27	93.7	8.91 ± 0.59	93.8	0.744
	*P. communis* cv. Shygys	No. 1	21.9 ± 1.01	−	21.1 ± 2.69	96.1	24.4 ± 3.25	111	0.325
		No. 2		a	13.4 ± 1.55 b	61.0	10.3 ± 0.84 c	46.8	<0.001
CAT	*M. domestica* cv. Aport	No. 1	2.60 ± 0.48	c	5.16 ± 0.38 b	199	7.51 ± 0.79 **a**	289	<0.001
		No. 2		c	921 ± 27.6 b	355×	1 849 ± 230 **a**	713×	<0.001
	*P. communis* cv. Shygys	No. 1	1 742 ± 88.5	b	2 333 ± 48.2 ab	134	5 385 ± 404 **a**	309	<0.01
		No. 2		b	2 920 ± 165 a	168	1 290 ± 66.1 **c**	74.0	<0.001
GR	*M. domestica* cv. Aport	No. 1	1.03 ± 0.05	−	0.76 ± 0.18	73.4	0.80 ± 0.07	77.5	0.051
		No. 2		a	0.22 ± 0.02 b	21.5	0.27 ± 0.02 b	26.5	<0.01
	*P. communis* cv. Shygys	No. 1	0.65 ± 0.04	c	1.10 ± 0.04 b	170	1.52 ± 0.13 a	235	<0.001
		No. 2		b	0.76 ± 0.04 b	117	1.26 ± 0.03 a	195	<0.01
SOD	*M. domestica* cv. Aport	No. 1	708 ± 28.2	c	884 ± 24.9 b	125	1 203 ± 78.8 a	170	<0.001
		No. 2		−	674 ± 19.3	95.3	736 ± 54.3	104	0.206
	*P. communis* cv. Shygys	No. 1	237 ± 7.67	a	58.6 ± 4.08 b	24.7	22.2 ± 2.09 c	9.36	<0.01
		No. 2		b	243 ± 14.8 ab	103	266 ± 6.13 a	112	<0.05

Notes: Cv.—cultivar; BP—biopreparation; Ctrl—control; APX—ascorbate peroxidase; CAT—catalase; GR—glutathione reductase; SOD—superoxide dismutase.

## Data Availability

Data available upon justified request to the corresponding author.

## Supplementary Materials

The following supporting information can be downloaded at: https://www.mdpi.com/article/10.3390/plants13111431/s1, Table S1: Chlorophyll pigment content in untreated plants (mg mL^−1^).
