# Supplementary material for: In Vitro Approbation of Microbial Preparations to Shield Fruit Crops from Fire Blight: Physio-Biochemical Parameters
Source: Plants (Basel). 2024 May 22;13(11):1431. doi: 10.3390/plants13111431 (PMC11174909; doi:10.3390/plants13111431)
Supplement: Supplementary file 1 [file plants-13-01431-s001.zip › plants-2976386-supplementary.pdf]

Table S1. Chlorophyll pigments and carotenoids content in untreated plants (mg mL^{-1}). Different letters (a, b, c) within one parameter show the significant difference.

**Parameter**	***M. sieversii*** **cv. KG10** Blight-resistant	***M. domestica*** **cv. Aport** Blight-susceptible	**% to resistant**	***p*-value**	***P. pyraster*** **cv. Wild** Moderately resistant	***P. communis*** **cv. Shygys** Blight-susceptible	**% to resistant**	***p*-value**
Chl a	20.3 ± 0.77	17.5 ± 1.76	86.1	**0.065**	15.1 ± 2.11	16.4 ± 1.4	109	**0.413**
Chl b	9.67 ± 0.63 **a**	7.57 ± 0.97 **b**	78.2	< 0.05	6.34 ± 0.76	6.71 ± 0.86	106	**0.602**
Car	5.10 ± 0.10 **a**	4.59 ± 0.04 **b**	90.1	< 0.01	5.31 ± 0.08	4.99 ± 0.34	94.0	**0.186**
Chl a/b	2.10 ± 0.06 **b**	2.32 ± 0.06 **a**	110	< 0.05	2.38 ± 0.05	2.46 ± 0.11	103	**0.317**
Chl (a+b)/Car	5.88 ± 0.39	5.44 ± 0.54	92.6	**0.323**	4.03 ± 0.47	4.64 ± 0.18	115	**0.106**

Notes: Cv. – cultivar; *Chl a* – chlorophyll a; *Chl b* – chlorophyll b; *Car* – carotenoids.
